# Climate change and health: recent progress

**DOI:** 10.2471/BLT.14.148130

**Published:** 2014-11-01

**Authors:** Alistair Woodward

**Affiliations:** a School of Population Health, University of Auckland, Private Bag 92019, Auckland, 1050, New Zealand.

It is almost 20 years since the Intergovernmental Panel on Climate Change (IPCC) published its second assessment report; the first to include a chapter on human health. It is almost eight years since the IPCC and Al Gore were jointly awarded the Nobel Peace Prize, for work on climate change. What progress has since been made on the climate change and health agenda?

Most relevant research in the 1990s and 2000s focused on the effects of climate change on health, without any detailed assessment of the effects of changes in other important drivers of health. However, we now have studies that model the effects of climate change and possible future social, demographic and economic trends. We therefore have a much more detailed and policy-relevant picture of future risks and vulnerabilities. For example, a recent risk assessment of the effects of climate change on selected causes of mortality, cites several such studies that focused on undernutrition.^1^

We now have some idea of where the largest risks lie – i.e. the risks posed by the high-end climate scenarios in which the mean global temperature rises to about 4 °C above its pre-industrial values by 2100. The IPCC’s fifth assessment report^2^ argues that we should take such risks seriously for three main reasons. First, emissions of greenhouse gases show annual growth that approximates the result of the IPCC’s worst scenario model. Second, in the same scenario, the climate does not stabilize in 2100 but carries on warming until the global mean temperature exceeds its pre-industrial levels by about 10–12 °C.^3^ (These projections are subject to enormous uncertainties.) Third, the predicted effects of such extreme global heating would probably exceed the limits of human adaptation. For example, an exponential rise in the risk of catastrophic weather events, exposure of essential food crops to lethal temperatures, and unprecedented periods of high temperatures and high humidity would overwhelm human adaptive capacity.^4^

Like the associated risks, the opportunities for addressing climate change are better characterized now than they were even a decade ago. If we replaced electricity produced in coal-burning power stations with electricity produced in hydro-electric plants, wind turbines or power stations fuelled with natural gas, we would generate fewer greenhouse gases and reduce exposure to air pollution – with major health benefits.^2^ It has been estimated that, by reducing black carbon and methane emissions, we could not only halve the projected increase in global temperatures by 2050 but also avoid approximately 2.4 million premature deaths per year.^5^

It is now clear that climate-change mitigation and economic development can be achieved simultaneously. Implementing some economic policies could probably take us most of the way to the so-called two-degree path – in which the global mean temperature increases by no more than 2 °C above its pre-industrial levels by 2100 – while supporting employment, social development and good health.^6^ For example, compact cities that are built around an effective system for mass public transport might produce a relatively small carbon footprint and relatively low levels of pollution while promoting high levels of physical activity and productivity.

Twenty years ago the association between health and climate change was the concern of a small number of scientists. Now climate change is recognized by the world’s international agencies as an unprecedented threat to human health and well-being. In his opening address to the first World Health Organization Conference on Climate Change and Health, held in Geneva in August 2014, Jim Yong Kim – President of the World Bank – acknowledged that climate change, health and international development are inseparable. Although recognition of this kind is a significant sign of progress on the climate change and health agenda, substantial action on climate change is likely to be achieved only with major changes to the world’s economy.^7^

Concerted international action will require politicians who are confident of local support and this is one of the most important reasons for all citizens – including health professionals – to think globally and act locally. If health professionals can lead by example, they may not only cut costs and reduce environmental risks but also help to shift social norms and empower politicians to act effectively, with potentially immense long-term benefits.
